# Supplementary material for: Structural inhibition of dynamin-mediated membrane fission by endophilin
Source: eLife. 2017 Sep 21;6:e26856. doi: 10.7554/eLife.26856 (PMC5663480; doi:10.7554/eLife.26856)
Supplement: Figure 2—source data 1. [file elife-26856-fig2-data1.docx]

**Source data for Figure 2-Figure Supplement 1**

Endophilin intensity on tube increases with increased endophilin concentration in solution.

| -Endo | +Endo (0.5x) | +Endo (1x) |
| --- | --- | --- |
| N/A | 172  204  182  453  418  296  457  337  468  381  296 | 437  424  1123  804  328  494  790  687  48  34  103  133  597  715 |
