# Supplementary material for: Structural inhibition of dynamin-mediated membrane fission by endophilin
Source: eLife. 2017 Sep 21;6:e26856. doi: 10.7554/eLife.26856 (PMC5663480; doi:10.7554/eLife.26856)
Supplement: Figure 4—source data 1. [file elife-26856-fig4-data1.docx]

**Source data for Figure 4F**

Distribution of dynamin event durations for non-transfected (NT) and endophilinA2-TagRFP overexpressing cells.

| **EndoA2 OE** | **NT** |
| --- | --- |
| 100 100 100 100 100 100 100 100 100 100 100 100 100 100 100 100 100 100 100 100 100 100 100 100 100 100 100 100 100 100 100 100 100 100 100 100 100 100 100 100 100 100 100 100 100 100 100 100 100 100 100 100 100 100 100 100 100 100 100 100 100 100 100 100 100 100 100 100 100 100 100 100 100 100 100 100 100 100 100 100 100 100 100 100 100 100 100 100 100 100 100 100 100 100 100 100 100 100 100 100 100 100 100 100 100 100 100 100 100 100 100 100 100 100 100 100 100 100 100 100 100 100 100 100 100 100 100 100 100 100 100 100 100 100 100 100 100 100 100 100 100 100 100 100 100 100 100 100 100 100 100 100 100 100 100 100 100 100 100 100 100 100 16.13873204 16.80918629 2.79765025 9.079589366 9.681931455 9.610915745 8.745104878 4.7053375 10.46369869 6.158633584 3.306251696 4.539845016 68.70639988 8.391999104 4.565797004 | 16.89095454 4.673838592 16.70130639 6.310073187 3.003883619 9.957152207 4.100945901 5.123677508 14.38705049 2.568405395 22.7019486 4.821546489 3.738744661 19.7663887 3.961873853 20.46001469 13.30881909 6.704561348 5.505319164 4.875940135 4.368912783 3.93442848 14.75996064 5.111805189 3.252741842 3.542273063 15.98376078 16.37485617 8.904081175 55.36389952 5.246250335 8.841485794 20.0791764 8.153349339 9.166959919 4.160285458 3.39977181 4.775446083 4.366884392 13.54106845 4.346362864 12.01889223 3.843901081 9.157364631 24.94941416 2.866196482 15.01194158 5.159583551 29.10830316 48.05309429 28.72256345 31.25619559 32.21721152 12.89260298 6.224032973 8.99978839 6.906845495 23.54979954 3.538524684 5.448213267 5.187504254 9.11400476 3.818689351 3.127104471 3.886360207 4.898558016 3.848060255 36.83643483 2.427790586 7.295495238 3.29564618 29.97168824 4.158673382 40.96093286 72.364663 19.49354538 9.931479249 4.719043483 4.303012755 5.233368905 6.072421983 15.50180974 4.601562226 23.30514171 3.490430994 11.97504385 6.005051965 22.33271793 4.112244817 12.25290222 5.943762478 7.833150674 3.726201234 5.605163681 14.65697396 31.73826716 5.279981027 5.92390842 4.698983943 5.86577936 4.771626802 4.933670467 4.503806975 9.173763096 15.07624745 17.94109997 6.620590098 5.140454751 4.715443565 7.707367258 15.5645036 20.74119986 2.539791368 5.241398624 12.01689345 15.57978182 8.921593628 19.3660503 19.66127079 20.77035725 18.74023736 33.4287774 4.395573776 13.24088286 4.546149172 6.820307344 14.20916389 3.662566412 19.59527016 28.9898908 12.75838352 6.528186136 3.572036676 5.823637496 6.442332224 3.63170416 7.185341452 8.504757844 78.53862216 20.60026688 6.613614944 3.335776208 12.83814948 10.35464521 19.61296659 22.88890104 19.29453332 75.05928488 6.975515288 20.26680022 6.101571032 4.132309716 14.1364055 4.772396792 2.736891076 18.28702897 2.847915216 27.80532211 7.268739436 11.00925574 27.37369881 3.204369644 18.10611037 107.526288 10.7343645 43.23275828 3.608288852 10.25318434 13.642134 7.3889397 4.172832876 20.64529678 51.23346527 7.022285824 22.13926484 41.63521076 9.808114544 5.881929468 4.76421014 8.723894288 12.33117588 3.358953208 3.078230768 3.503102368 |

**Unpaired t test pit duration**

|  | Data Set-A |
| --- | --- |
| Table Analyzed  Column B vs. Column A  Unpaired t test P value P value summary Significantly different (P < 0.05)? One- or two-tailed P value? t, df  How big is the difference? Mean ± SEM of column A Mean ± SEM of column B Difference between means 95% confidence interval R squared (eta squared)  F test to compare variances F, DFn, Dfd P value P value summary Significantly different (P < 0.05)? | Figure 4F  NT vs. EndoA2 OE   <0.0001 **** Yes Two-tailed t=36.96 df=359   92.56 ± 1.874, n=177 13.34 ± 1.083, n=184 -79.22 ± 2.144 -83.44 to -75.01 0.7918   2.882, 176, 183 <0.0001 **** Yes |
