# Supplementary material for: Structural inhibition of dynamin-mediated membrane fission by endophilin
Source: eLife. 2017 Sep 21;6:e26856. doi: 10.7554/eLife.26856 (PMC5663480; doi:10.7554/eLife.26856)
Supplement: Figure 4—source data 2. [file elife-26856-fig4-data2.docx]

**Source data for Figure 4H**

Quantification of transferrin fluorescence in non-transfected cells (NT), cells overexpressing endophilinA2-TagRFP (tot), cells with high (>10 000) and low (<10 000) endophilinA2-TagRFP levels, and cells co-overexpressing dynamin 2 and endophilinA2-GFP (Dyn2 OE)

| **NT** | **tot** | **>10 000** | **<10 000** | **Dyn2 OE** |
| --- | --- | --- | --- | --- |
| 29718 12954 17205 19805 25371 11275 15842 26281 21159 12879 17809 15570 23791 21449 21205 29262 16259 21962 15098 14744 10899 | 12636 9898 15943 15929 20813 22927 21966 14146 6333 18595 20630 13862 8589 11600 30848 24351 18742 6905 6626 24955 16886 8024 5625 7284 16552 9884 19966 9338 12385 36585 21875 15232 7213 7969 8162 5836 5851 8579 9487 6918 6894 7640 8643 10788 7508 15213 15446 25812 | 12636 9898 15943 14146 6333 11600 18742 6905 6626 8024 7284 9884 7213 7969 5836 5851 6918 6894 7640 10788 7508 15213 15446 25812 | 15929 20813 22927 21966 18595 20630 13862 8589 30848 24351 24955 16886 5625 16552 19966 9338 12385 36585 21875 15232 8162 8579 9487 8643 | 9501 11422 20754 9731 7626 21865 17609 14365 20416 32534 22988 13623 11189 9464 19876 21518 26177 15271 13108 13673 10603 20551 24264 23592 22798 15017 27386 22970 25682 11638 14298 28973 16477 30561 19621 22764 18520 16705 26108 24044 29142 23296 8643 10788 7508 15213 15446 25812 |

**Unpaired t test of "NT" vs. "tot"**

|  | Data Set-A |
| --- | --- |
| Table Analyzed  Column A vs. Column B  Unpaired t test P value P value summary Significantly different? (P < 0.05) One- or two-tailed P value? t, df  How big is the difference? Mean ± SEM of column A Mean ± SEM of column B Difference between means 95% confidence interval R squared  F test to compare variances F,DFn, Dfd P value P value summary Significantly different? (P < 0.05) | Figure 4H  NT vs. tot   0.0046 ** Yes Two-tailed t=2.936 df=67   19073 ± 1223, n=21  13831 ± 1051, n=48 5242 ± 1786 1678 to 8806 0.1140   1.689, 47, 20 0.2030 ns No |

**Unpaired t test of "NT" vs. ">10 000****"**

|  | Data Set-A |
| --- | --- |
| Table Analyzed  Column A vs. Column C  Unpaired t test P value P value summary Significantly different? (P < 0.05) One- or two-tailed P value? t, df  How big is the difference? Mean ± SEM of column A Mean ± SEM of column C Difference between means 95% confidence interval R squared  F test to compare variances F,DFn, Dfd P value P value summary Significantly different? (P < 0.05) | tot_endo > 10000  NT vs. >10 000   < 0.0001 **** Yes Two-tailed t=5.473 df=43   19073 ± 1223, n=21  10463 ± 1011, n=24 8610 ± 1573 5438 to 11783 0.4106   1.281, 20, 23 0.5638 ns No |

**Unpaired t test of "NT" vs.** **"<10 000"**

|  | Data Set-A |
| --- | --- |
| Table Analyzed  Column A vs. Column D  Unpaired t test P value P value summary Significantly different? (P < 0.05) One- or two-tailed P value? t, df  How big is the difference? Mean ± SEM of column A Mean ± SEM of column D Difference between means 95% confidence interval R squared  F test to compare variances F,DFn, Dfd P value P value summary Significantly different? (P < 0.05) | Figure 4H  NT vs. <10 000   0.3644 ns No Two-tailed t=0.9168 df=43   19073 ± 1223, n=21  17199 ± 1583, n=24 1874 ± 2044 -2248 to 5996 0.01917   1.916, 23, 20 0.1462 ns No |
