# Supplementary material for: Structural inhibition of dynamin-mediated membrane fission by endophilin
Source: eLife. 2017 Sep 21;6:e26856. doi: 10.7554/eLife.26856 (PMC5663480; doi:10.7554/eLife.26856)
Supplement: Source code file 1. — The code determines the variables a and τ based on the experimental data, for a fit to a*(1-exp(-t/τ)). [file elife-26856-code.docx]

**Matlab code for fitting of data for Fig. 2L**

%defining variables (adapt if more or less curves)

ratio0 = xlsread('excelforfit.xlsx','ratio_0')

ratio0dot5 = xlsread('excelforfit.xlsx','ratio_0dot5')

ratio1 = xlsread('excelforfit.xlsx','ratio_1')

%make fit (take out those not needed today)

s=fitoptions('Method', 'NonlinearLeastSquares',...

'Lower', [0 0],...

'Upper', [1.0001 Inf]);

g=fittype('a*(1-exp(-x./tau))','coeff',{'a','tau'} ,...

'options',s);

[fitfun_0, gof_0]=fit(ratio0(:, 1), ratio0(:,2), g);

[fitfun_0dot5, gof_0dot5]=fit(ratio0dot5(:, 1), ratio0dot5(:,2), g);

[fitfun_1, gof_1]=fit(ratio1(:, 1), ratio1(:,2), g);

%[fitfun_2, gof_2]=fit(ratio2(:, 1), ratio2(:,2), g);

%[fitfun_4, gof_4]=fit(ratio4(:,1), ratio4(:,2), g);

%[fitfun_4contr5tot, gof_4contr5tot]=fit(ratio4contr5tot(:,1), ratio4contr5tot(:,2), g);

s=fitoptions('Method', 'NonlinearLeastSquares',...

'Lower', [0 3],...

'Upper', [1.0001 Inf])

g=fittype('a*(1-exp(-x./tau))','coeff',{'a','tau'} ,...

'options',s);

%create vectors with fit function

xrange=0:1:300;

y_0=fitfun_0(xrange);

y_0dot5=fitfun_0dot5(xrange);

y_1=fitfun_1(xrange);

%y_2=fitfun_2(xrange);

%y_4=fitfun_4(xrange);

%y_4contr5tot=fitfun_4contr5tot(xrange);

%create txt files for each vector

xrange=xrange'; save xrange.txt xrange -ASCII

save y_0.txt y_0 -ASCII

save y_0dot5.txt y_0dot5 -ASCII

save y_1.txt y_1 -ASCII

% figure('position',[200 300 515 490]);

figure('position',[200 300 315 300])

xrange=0:1:300;

y_0=fitfun_0(xrange);

y_0dot5=fitfun_0dot5(xrange);

y_1=fitfun_1(xrange);

%y_2=fitfun_2(xrange);

%y_4=fitfun_4(xrange);

%y_4contr5tot=fitfun_4contr5tot(xrange);

%insert graphs of fits into figure

plot(xrange, y_0,...

'Color', [0.153 .565 0.741],...

'Linewidth',2);

hold on

plot(xrange, y_0dot5,...

'Color', [0.533 0.647 0.243],...

'Linewidth',2);

hold on

plot(xrange, y_1,...

'Color', [0.898 0.773 0.329],...

'Linewidth',2);

hold on

%plot(xrange, y_2,...

% 'Color', [.451 .102 .255],...

% 'Linewidth',2);

%hold on

%plot(xrange, y_4,...

% 'Color', [.329 .118 .169],...

% 'LineWidth',2);

%hold on

%plot(xrange, y_4contr5tot,'--',...

% 'Color', [.329 .118 .169],...

% 'LineWidth',1.5);

hold on

%insert measured dots into same graph

plot(ratio0(:, 1),ratio0(:, 2),'s',...

'Linewidth',1,...

'MarkerEdgeColor','k',...

'MarkerFaceColor',[0.153 .565 0.741],...

'MarkerSize',10);

hold on

plot(ratio0dot5(:, 1),ratio0dot5(:, 2),'s',...

'Linewidth',1,...

'MarkerEdgeColor','k',...

'MarkerFaceColor',[0.533 0.647 0.243],...

'MarkerSize',10);

hold on

plot(ratio1(:, 1),ratio1(:, 2),'s',...

'Linewidth',1,...

'MarkerEdgeColor','k',...

'MarkerFaceColor',[0.898 0.773 0.329],...

'MarkerSize',10);

%hold on

%plot(ratio2(:, 1),ratio2(:, 2),'s',...

% 'Linewidth',1,...

% 'MarkerEdgeColor','k',...

% 'MarkerFaceColor',[.557 .208 .341],...

% 'MarkerSize',10);

%hold on

%plot(ratio4(:, 1),ratio4(:, 2),'s',...

% 'Linewidth',1,...

% 'MarkerEdgeColor','k',...

% 'MarkerFaceColor',[.329 .118 .169],...

% 'MarkerSize',10);

%hold on

%plot(ratio4contr5tot(:, 1),ratio4contr5tot(:, 2),'O',...

% 'Linewidth',1.25,...

% 'MarkerEdgeColor',[.329 .118 .169],...

% 'MarkerFaceColor',[1 1 1],...

% 'MarkerSize',7);

hold on

%define colors etc. of graph borders

set(gca,'LineWidth',1.5);

set(gca, 'FontName', 'Trebuchet MS',...

'FontSize', 14,...

'FontWeight', 'b')

set(gca,'xcolor','k','ycolor','k')

%xlabel('Time, s');

%ylabel('Cumulative Probability of Fission');

xlim([-5 300]);

ylim([-.02 1.02]);

%print fit parameters

gof_0

gof_0dot5

gof_1

% gof_2

% gof_4

% gof_4contr5tot

fitfun_0

fitfun_0dot5

fitfun_1

%fitfun_2

%fitfun_4

%fitfun_4contr5tot
