# Supplementary material for: Structural inhibition of dynamin-mediated membrane fission by endophilin
Source: eLife. 2017 Sep 21;6:e26856. doi: 10.7554/eLife.26856 (PMC5663480; doi:10.7554/eLife.26856)
Supplement: Transparent reporting form [file elife-26856-transrepform.docx]

***eLife’s* transparent reporting form**

We encourage authors to provide detailed information *within their submission* to facilitate the interpretation and replication of experiments. If you have any questions, please contact us: [editorial@elifesciences.org](mailto:editorial@elifesciences.org).

**Sample-size estimation**

- You should state whether an appropriate sample size was computed when the study was being designed

There was no computer-based estimation of sample size.

- You should state the statistical method of sample size computation and any required assumptions

There was no statistical method involved in determining the sample size.

- If no explicit power analysis was used, you should describe how you decided what sample (replicate) size (number) to use

At least 10 replicas were used in general for the in-vitro experiments. For experiments where the analysis was automated, very large numbers were used (>100 events, with 3 independent cells for each condition).

Please outline where this information can be found within the submission (e.g., page numbers or figure legends), or explain why this information doesn’t apply to your submission:

See for each point below

**Replicates**

- You should report how often each experiment was performed

The number of replicas is indicated, where applicable, in the figure legend.

- You should include a definition of biological versus technical replication

Biological replicates are made with independent samples made on different days. Technical replicates are duplications of experiment done the same day with the same sample.

- The data obtained should be provided and sufficient information should be provided to indicate the number of independent biological and/or technical replicates

Details for each experiment, where applicable, are reported in the figure legend.

- If you encountered any outliers, you should describe how these were handled

There were no outliers for most of the experiments. The outliers from the automated analysis came from errors in the fitting, thus they were excluded from the graphs.

- Criteria for exclusion/inclusion of data should be clearly stated

See above.

- High-throughput sequence data should be uploaded before submission, with a private link for reviewers provided (these are available from both GEO and ArrayExpress)

No high-throughput sequence data was used.

Please outline where this information can be found within the submission (e.g., page numbers or figure legends), or explain why this information doesn’t apply to your submission:

**Statistical reporting**

- Statistical analysis methods should be described and justified

Data and statistical analysis is described in the text (pag.10-12; 28-29) and statistical reports are available in the source data files and in Fig. 1 and 4.

- Raw data should be presented in figures whenever informative to do so (typically when N per group is less than 10)

For several in vitro experiments the raw data are plotted directly in the figure (Fig. 2E,H,L). Source files are uploaded for all graphs where averages and standard deviations are plotted in the graph. Where applicable, statistical reports are available in the respective source file.

- For each experiment, you should identify the statistical tests used, exact values of N, definitions of center, methods of multiple test correction, and dispersion and precision measures (e.g., mean, median, SD, SEM, confidence intervals; and, for the major substantive results, a measure of effect size (e.g., Pearson's r, Cohen's d)

Full results of statistical analysis are available in source data files. The definitions of the represented error bars are indicated in the respective figure legends.

- Report exact p-values wherever possible alongside the summary statistics and 95% confidence intervals. These should be reported for all key questions and not only when the p-value is less than 0.05.

*p*-values are reported in the statistical reports inside the source files.

Please outline where this information can be found within the submission (e.g., page numbers or figure legends), or explain why this information doesn’t apply to your submission:

(For large datasets, or papers with a very large number of statistical tests, you may upload a single table file with tests, Ns, etc., with reference to page numbers in the manuscript.)

**Additional data files (“source data”)**

- We encourage you to upload relevant additional data files, such as numerical data that are represented as a graph in a figure, or as a summary table

Source data files for graphs visualizing averages and standard deviations are provided.

- Where provided, these should be in the most useful format, and they can be uploaded as “Source data” files linked to a main figure or table

Source data files for graphs are provided

- Include model definition files including the full list of parameters used

NA

Include code used for data analysis (e.g., R, MatLab)

Matlab code is provided in separate files.

Avoid stating that data files are “available upon request”

Please indicate the figures or tables for which source data files have been provided:

Figure 2K

Figure 2-Figure Supplement1

Figure 4F,H
